# Adherence to the Japanese Diet is Associated with Lower Risk of Geriatric Depression: A Prospective-Cohort Study Based on the New Integrated Suburban Seniority Investigation (NISSIN) Project

**DOI:** 10.1016/j.jnha.2025.100689

**Published:** 2025-09-29

**Authors:** Ho Chen, Eri Maeda, Wen Hao, Kenji Wakai, Satoe Okabayashi, Shigekazu Ukawa, Akiko Tamakoshi

**Affiliations:** a School of Medicine, Hokkaido University. Kita15, Nishi 7, Kita-ku, Sapporo 060-8638, Japan; b Department of Psychiatry and Behavioral Health, The Ohio State University Wexner Medical Center. 1676 Upham Dr, Columbus, OH 43210, United States of America; c Department of Public Health, Faculty and Graduate School of Medicine, Hokkaido University. Kita15, Nishi 7, Kita-ku, Sapporo 060-8638, Japan; d Department of Preventive Medicine, Nagoya University Graduate School of Medicine. 65 Tsurumai-cho, Showa-ku, Nagoya 466-8550, Japan; e Agency for Health, Safety and Environment, Kyoto University, Yoshida-Honmachi, Sakyo-Ku, Kyoto 606-8501, Japan; f Osaka Metropolitan University Graduate School of Human Life and Ecology. 3-3-138, Sugimoto, Sumiyoshi-Ku, Osaka 558-8585, Japan

**Keywords:** Japanese diet, Geriatric depression, Prospective cohort, Fish and shellfish, Green-yellow vegetable, Soybean-derived product

## Abstract

**Objectives:**

Geriatric depression is an increasingly important public health issue in an aging society. However, while Japan boasts one of the world’s highest and healthiest life expectancies at birth, there was no prospective cohort study dedicated to the specific association between the Japanese diet and geriatric depression. Thus, we aimed to examine this relationship and assess whether such association extends beyond better physical health secondary to higher diet quality.

**Design, setting, participants, and measurements:**

Our study utilized the New Integrated Suburban Seniority Investigation (NISSIN) Project, which is an age-specific prospective cohort study that recruited residents of Nisshin City, Japan who were about to reach 65 years of age between 1996 and 2005. We measured the adherence to the Japanese diet of 1620 elderly Japanese individuals (827 male and 793 female) with a modified version of the Japanese Diet Index (JDI) and assessed the development of geriatric depression with the Geriatric Depression Scale 15 items questionnaire when they reached 70 years of age.

**Results:**

A total of 135 individuals developed geriatric depression at 70 years of age. After adjusting for major confounding factors, those within the highest group of adherence to the Japanese diet had significantly reduced risk of developing geriatric depression (adjusted odds ratio [aOR] = 0.525, 95% confidence interval [CI]: 0.286 – 0.962) when compared to those with the lowest adherence, and each point received on the JDI was also associated with reduced risk of geriatric depression (aOR = 0.900, 95% CI: 0.816−0.992). Dietary item-wise analyses showed that fish and shellfish (p = 0.024), green-yellow vegetable (p = 0.003), and soybean-derived products (p = 0.001) were significantly associated with lower risk of geriatric depression.

**Conclusion:**

Adherence to the Japanese diet, especially those rich in green-yellow vegetables, soybean-derived products, and fish and shellfish, may be protective against geriatric depression.

## 1 Introduction

As the saying goes, “You are what you eat.” Diet plays a pivotal role in one’s cultural identity as well as physical and mental health. Among Western scholars, this association is most widely studied in the Mediterranean diet, and it was found that higher adherence to this diet is associated with lower risks of age-associated non-communicable diseases (NCDs) [1]. On the other hand, Japan boasts one of the world’s highest and healthiest life expectancies at birth [2]. Thus, unsurprisingly, the effect of the Japanese diet on the health of its population has also become a popular topic of research.

The unique characteristic that distinguishes the Japanese dietary pattern is the reduced intakes of red meat, milk, and dairy products as well as the higher intakes of fish and shellfishes, soybeans, non-starchy vegetables, and non-sweetened beverages [3,4]. The Japanese diet is partially similar to the Mediterranean diet and is generally considered to be beneficial to the physical health [5]. Previous studies have recognized that adherence to the Japanese diet is associated with a decreased risk of all-cause and cardiovascular disease (CVD) mortality among Japanese adults [6]. Likewise, it was found that the Japanese diet is associated with lower risk of developing cognitive decline, and some researchers had suggested that the lower concentrations of gut microbial metabolites associated with the Japanese diet might have been responsible for this effect through the diet-microbiome-gut-brain axis [7]. Overall, it appears that the health-promoting characteristics of the Japanese diet have been well established. In this study, we aim to understand whether such beneficial effect can be extended to another important components of health that is geriatric depression.

Geriatric depression is a crucial topic in public health, as it closely interferes with the general wellness of elders. Previous research estimated that among community dwelling elders in Japan, 33.5% reported screening-based mild depression and 11.3 % reported severe depression [8]. It was also found that up to 50% of patients with chronic disease have depression, which can impact the treatment and prognosis of such existing comorbidity that presents at high prevalence among the elderly population [9]. Combined with a rapidly aging of modern society, understanding the influence of diet on the development of geriatric depression is gaining increasing importance. However, while previous studies have found that higher diet quality is associated with lower depression risk [10], no previous cohort study has specifically evaluated the association between the Japanese diet and geriatric depression in a Japanese population. Most existing studies exploring the diet of Japanese people and its association with geriatric depression were cross-sectional, and huge heterogeneity exists from the scoring methods of dietary pattern to their results [11,12]. A prospective cohort study by Okubo et al. found no association between diet quality and incidences of depression after adjusting for NCDs in a Japanese population [13]. However, their study did not measure the adherence to a Japanese diet, and it was neither age-specific nor did they study geriatric depression specifically.

This prompted us to approach this topic by utilizing the data collected through the New Integrated Suburban Seniority Investigation (NISSIN) Project, which is an age-specific prospective cohort [14]. We measured the adherence to the Japanese diet through a modified version of the Japanese Diet Index (JDI) [7]. We then assessed the extent of association between the Japanese diet and geriatric depression, especially after adjusting for history of NCDs and other lifestyle factor, to evaluate whether such association extends beyond better physical health secondary to higher diet quality.

## 2 Methods

### 2.1 Study design and participant selection

This study utilized the NISSIN project, with details of the study design noted elsewhere [14]. From 1996 to 2005, residents of Nisshin City, Japan who were about to reach 65 years of age in each year were invited to participate in health check-ups and completed a baseline self-administered questionnaire that included items on demographic and lifestyle characteristics, depression screening through the Geriatric Depression Scale 15 items (GDS-15) questionnaire [15], and a self-administrated food frequency questionnaire (FFQ) [16]. Follow-up health check-ups and questionnaires were conducted again when the participants reached 70 years of age. This study was approved by the Ethics Commitee of Hokkaido University Graduate School of Medicine (Medicine 14-037).

**Fig. 1:**
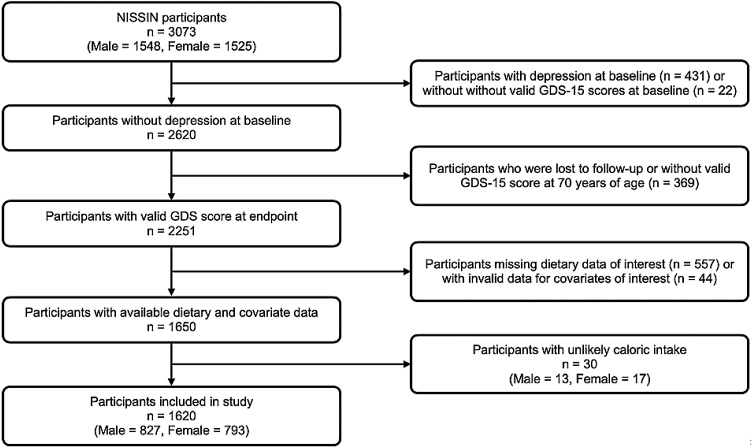
Participant inclusion and exclusion flowchart.

A total of 3073 participants consented to the participation of the study (1548 men and 1525 women). Participants with depression at baseline (n = 431), as defined by a GDS-15 score of 7 or above [17], or participants without valid GDS-15 scores at baseline (n = 22), were further excluded. Participants who were lost to follow-up or without valid GDS-15 score at 70 years of age were also excluded (n = 369). Those that were missing dietary data of interest (n = 557) and those with invalid data for covariates of interest (n = 44) were excluded. Finally, we excluded participants with unlikely caloric intakes (male n = 13, female n = 17). Likely caloric intakes were defined as 500–3500 kcal/day for female and 800–4000 kcal/day for male [18]. A total of 1620 individuals were subsequently included in this study, which consists of 827 male and 793 female. Detailed breakdown of this selection process is shown in Fig. 1.

### 2.2 Measurement of primary outcome

The primary outcome of this study was the development of geriatric depression at 70 years of age, which was defined as a GDS-15 score of 7 or above. While older studies have suggested a cut-off score of 6 for the GDS-15 [19], more recent studies showed that a cut-off score of 7 yields a higher value of Youden Index [17], and such this cut-off value was chosen.

### 2.3 Measurement of dietary adherence

Assessment of participant’s dietary intake was carried out using data collected from the self-administered FFQ at baseline [16]. The FFQ included 97 dietary items, which the participants were asked to report the average intake frequency of each food in reference to the standard portion sizes within the year prior to the survey. The average daily consumption amounts in grams were then calculated in a validated method by deconstructing dishes into dietary items and multiplying the intake frequency by the given portion size [20]. For dietary items with no available data estimating the daily consumption amount by the FFQ, such as miso and pickles, participants’ reported intake frequencies per day were used instead. In addition, coffee consumption was calculated as milliliters per day by multiplying the participants reported intake frequency (number of cups per day, week, or month) with cup size (200 mL, 250 mL, or 300 mL). Previous study has showed that over a timespan of five years, elderly participants aged 64–85 years exhibited similar and stable dietary pattern between baseline and endpoint [21]. Likewise, it was assumed that the diet pattern of our participants remained overall similar over the timespan of the study.

Adherence to the Japanese diet was calculated based on the 12-component revised JDI (rJDI_12_) [7]. Contrary to the other versions of the JDI including coffee as a non-beneficial component [22], the rJDI_12_ included eleven beneficial components (rice, miso, fish and shellfish, green-yellow vegetables, seaweed, pickles, soybean-derived food, fruits, mushroom, green tea, and coffee) and one non-beneficial component (beef and pork) due to evidence supporting the beneficial effects of coffee [7]. We therefore applied rJDI_12_ to our study as previous studies had also shown protective traits against depression when coffee was consumed moderately [23]. However, due to the lack of available data in the NISSIN study, we replaced beef and pork with general meat category and removed the green tea item from the rJDI12, which resulted in a 11-component revised JDI (rJDI_11_) that included ten beneficial components and one non-beneficial component.

For each of the ten beneficial dietary items in this rJDI_11_, the participants were assigned 1 point if their daily consumption of the item was equal to or greater than the sex-specific median. For the less-beneficial dietary item, participants were assigned 1 point if their daily consumption was less than the sex-specific median. The total scores were then tallied for each participant, which would range from 0 to 11 and indicate overall adherence to the Japanese diet. Participants were then divided into four groups according to their rJDI_11_ scores. Those with a score of 0–4, 5–6, 7, and 8–11 were categorized to as Q1, Q2, Q3, and Q4, respectively, to represent increasing adherence to the Japanese diet. This cutoff was selected to distribute the participants as evenly as possible.

### 2.4 Selection of covariates

Better diet quality has been shown to closely correlate with overall healthier lifestyles, including higher physical fitness, less alcohol use, and less tobacco use [[24], [25], [26]]. Thus, a wide spectrum of measurements of lifestyle factors were included to adjust for possible confounding effects.

Baseline characteristics considered in this study included sex, daily caloric intake calculated through the FFQ estimation, enrollment year, and Body mass index (BMI) (Low: < 18.5, Medium: ≥ 18.5 and < 25, High: ≥ 25). Other lifestyle factors considered include participants’ self-reported exercise level (hardly-never, less than once per week, equal or more than once per week), tobacco use (never-smoker, ≤ 15 pack-year, > 15 pack-year), alcohol use (never drinker, current drinker), highest education level (elementary school or junior high school, high school or junior college/specialized college, university or higher), family members living within same household, current marital status, and daily sleep time in hours. Also, as major NCDs were found to have psychosomatic features [8], self-reported medical history of hypertension (HTN), hyperlipidemia (HLD), coronary artery disease (CAD), cerebral vascular accident (CVA), or type II diabetes mellitus (DM) at baseline were considered as well.

### 2.5 Statistical analysis

Geriatric depression outcomes and baseline characteristics were compared between Q1, Q2, Q3 and Q4 to detect whether there was any underlying difference. We also compared the baseline characteristics between the 1620 participants included in the study and the 1000 participants that had no depression at baseline but were excluded due to not meeting the inclusion criteria of this study to assess for possible bias in the selection of our study participants. Statistical differences were assessed using a Chi-squared test for categorical measurements as well as rJDI_11_ scores. For continuous measurements, statistical differences were detected with Kruskal–Wallis test. Consumption amount for each dietary item on the rJDI_11_ were also compared among the four groups to assess whether the rJDI_11_ provided a reasonable estimation of overall adherence to the Japanese diet, and Cuzick’s test for trend was utilized to assess the significance.

To assess whether adherence to the Japanese diet is associated with geriatric depression after adjusting for possible confounding factors, binary logistic regressions were conducted with the development of geriatric depression at 70 years of age as the outcome. rJDI_11_ groups as factors (Q2, Q3, or Q4 in reference to Q1), rJDI_11_ groups as continuous linear trend (in the order of Q1, Q2, Q3, and Q4), and the raw rJDI_11_ scores were treated as the primary independent variables. One crude and two multivariable logistic regression models were constructed for each of the primary independent variable. No adjustment was made to the crude model. Model 1 was adjusted for baseline characteristics and lifestyle factors including sex, enrollment year, estimated daily caloric intake, BMI (in reference to the Medium group), education level, tobacco use, alcohol use, marriage status, and exercise level. Model 2 was further adjusted for any reported medical history of NCDs (HTN, HLD, CAD, CVA, DM) in addition to Model 1. Logistic regressions for Model 1 were also conducted after stratification of baseline health status (i.e., with or without any NCDs). In addition, we used the more traditional cut-off score of 6 in the GDS-15 for depression within this participant group and conducted the analyses to assess the robustness of our findings.

Finally, the scoring for each dietary item of the rJDI_11_ were compared between those who did not develop depression and those who developed depression at 70 years of age to examine whether any specific dietary item included in the rJDI_11_ is associated with the development of geriatric depression. The differences between the groups were assessed by Chi-squared test. We also conducted binary logistic regressions with the development of geriatric depression at 70 years of age as the outcome and scoring for each dietary item as the primary independent variables. Adjustments for these regression models were made to reflect the crude model, Model 1, and Model 2 mentioned in the previous paragraph.

All reported p-values were two-sided, and a p-value of < 0.05 was considered statistically significant. All statistical analyses were performed using R version 4.3.2 (2023-10-31) [27].

## 3 Results

Based on their rJDI_11_ score, 482 participants were categorized as Q1 (29.8%), 488 participants as Q2 (30.1%), 270 participants as Q3 (16.7%), and 380 participants as Q4 (23.5%) (Table 1). Participants in higher rJDI_11_ score groups were more likely to have higher exercise level (p = 0.001) at baseline, while most other baseline characteristics, including the GDS-15 score at baseline, were similar between the groups. Besides marital status (p = 0.021), there was no significant difference between participants included and excluded from the study (Supplementary Table S1). As shown in Table 2, we found that participants in higher rJDI_11_ score groups tend to have higher daily caloric intake (1588 kCal vs. 1763 kCal vs. 1986 kCal vs. 2252 kCal, p < 0.001). For all dietary item in the rJDI_11_, those in the higher rJDI_11_ score groups were likely to consume more than their counterparts in lower rJDI_11_ score groups, regardless of the beneficial status of the diet (p < 0.001 for all dietary items).

**Table 1:** Comparison of baseline characteristics between participants categorized by adherence to the Japanese diet using the 11-component revised Japanese Diet Index.

		Q1 (n = 482)	Q2 (n = 488)	Q3 (n = 270)	Q4 (n = 380)	
Characteristics	Categories	n (%) or Median (Q1−3rd Quartile)				P value
Sex	Female	242 (50.2)	234 (48.0)	129 (47.8)	188 (49.5)	0.876^a^
BMI	<18.5	19 (3.9)	26 (5.2)	13 (4.8)	13 (3.4)	0.487^a^
	≥18.5 and <25	347 (72.0)	365 (74.8)	205 (75.9)	281 (74.0)	
	≥25	116 (24.1)	97 (20.0)	52 (19.3)	86 (22.6)	
***Exercise Level***	***Hardly - Never***	***200 (41.5)***	***195 (40.0)***	***98 (36.3)***	***117 (30.8)***	***0.006***^a^
	***Less than once per week***	***37 (7.7)***	***35 (7.2)***	***33 (12.2)***	***45 (11.8)***	
	***Equal or more than once per week***	***245 (50.8)***	***258 (52.9)***	***139 (51.5)***	***218 (57.4)***	
Tobacco Use	Never smoker	263 (54.6)	256 (52.5)	148 (54.8)	223 (58.7)	0.427^a^
	≤15 pack-year	67 (13.9)	69 (14.1)	28 (10.4)	47 (12.4)	
	>15 pack-year	152 (31.5)	163 (33.4)	94 (34.8)	110 (29.0)	
Alcohol Use	Current drinker	232 (48.1)	228 (46.7)	142 (52.6)	165 (43.4)	0.137^a^
Education Level	Elementary school or junior high school	138 (28.6)	135 (27.7)	76 (28.1)	103 (27.1)	0.980^a^
	High school or junior college/specialized college	258 (53.5)	270 (55.3)	146 (54.1)	203 (53.4)	
	University or higher	86 (17.8)	83 (17.0)	48 (17.8)	74 (19.5)	
Marriage	Married	428 (88.8)	441 (90.4)	246 (91.1)	356 (93.7)	0.101^a^
Any Non-Communicable Diseases^c^		189 (39.2)	193 (39.6)	113 (41.9)	146 (38.4)	0.844^a^
Daily Sleep Time (hour)		7 (6–7.5)	7 (6–7.5)	7 (6–7.75)	7 (6–7.5)	0.654^b^
Members Within Same Household		1 (1–2)	1 (1–2)	1 (1–2)	1 (1–2)	0.703^b^
GDS-15 score		3 (1–4)	3 (1–4)	3 (1–4)	3 (2–4)	0.893^b^

GDS-15: Geriatric Depression Scale 15 items.

Characteristics with statistical significances between the groups are highlighted in bold italics. [a] P-values are calculated with Chi-square. [b] P-values are calculated with Kruskal-Wallis test. [c] Including any self-reported medical history of hypertension, hyperlipidemia, coronary artery disease, cerebral vascular accident, or type II diabetes mellitus.

**Table 2:** Comparison of daily intake for total calories and individual dietary items on the 11-component revised Japanese Diet Index between participants categorized by adherence to the Japanese diet.

		Q1 (n = 482)	Q2 (n = 488)	Q3(n = 270)	Q4 (n = 380)	
Dietary Item		Median (Q1−3rd Quartile)				P value^a^
Daily Caloric intake (kCal)		1588 (1309–1850)	1763 (1468–2103)	1986 (1700–2362)	2252 (1943–2626)	< 0.001
Beneficial	Coffee (mL per day)	200 (86–400)	200 (107–400)	250 (107–400)	250 (200–400)	< 0.001
	Rice (g per day)	33 (22–44)	36 (25–50)	38 (25−50)	45 (33–56)	< 0.001
	Miso (frequency per day)	0 (0–1)	1 (0–1)	1 (1 – 1)	1 (1 – 1)	< 0.001
	Fish and shellfish (g per day)	45 (31–60)	59 (40–92)	85 (57–129)	97 (72–139)	< 0.001
	Green-yellow vegetables (g per day)	56 (35–94)	82 (52–140)	116 (82–176)	156 (107–240)	< 0.001
	Seaweed (g per day)	1 (1–2)	3 (1–3)	3 (2–5)	5 (3–6)	< 0.001
	Pickles (frequency per day)	0 (0–1)	1 (0–1)	1 (0–1)	1 (0–1)	< 0.001
	Soybean-derived food (g per day)	48 (31–63)	65 (46–91)	82 (61–110)	101 (78–132)	< 0.001
	Fruits (g per day)	118 (64–184)	173 (99–255)	211 (137–310)	272 (197–362)	< 0.001
	Mushrooms (g per day)	4 (3–10)	10 (4–18)	10 (10–18)	18 (10–23)	< 0.001
Non-beneficial	Meat (g per day)	42 (25–58)	42 (26–66)	45 (29–65)	51 (31–78)	< 0.001

[a] P-values are calculated with Cuzick’s test for trend.

Among the 1650 individuals included in this study, 135 individuals (70 male and 65 female) developed geriatric depression at 70 years of age (Table 3). Participants in higher rJDI_11_ score groups were generally less likely to develop geriatric depression after 5 years. Logistic regression models (Table 3) showed that when comparing Q4 to Q1 within all participants, significantly reduced risks of developing geriatric depression were seen in all three models. When treating Q1 to Q4 as a continuous linear trend, significant association was only noted in the crude model, however the directionality of association preserved among all models. When treating the total rJDI_11_ score as the primary independent variable, higher rJDI_11_ score was consistently significantly associated with reduced risk of developing geriatric depression at endpoint across all three models. Stratification analyses showed that after adjusting for possible confounders in Model 1, no significant association was seen between adherence to the Japanese diet and the development of geriatric depression. Similarly, when using 6 as the alternative GDS-15 cutoff score for geriatric depression at endpoint, significant association was noted when comparing Q4 to Q1 across all models (Supplementary Table S2).

**Table 3:** Results of multiple logistic regression models reported in odds ratio (95% confidence interval) among all participants and participants stratified by their baseline non-communicable diseases status.

All Participants		Q1	Q2^a^	Q3^a^	Q4^a^	Trend^d^	rJDI-11 Score^e^
Number of participants		482	488	270	380	1620 (total)	
Number of cases (%)		53 (11.0)	39 (8.0)	23 (8.5)	20 (5.3)	135 (8.3)	
Crude		1	0.703 (0.455–1.085)	0.754 (0.451–1.260)	***0.450 (0.264 – 0.766)***	***0.792 (0.672 – 0.932)***	***0.882 (0.813−0.958)***
Model 1^b^		1	0.731 (0.468–1.142)	0.817 (0.474–1.408)	***0.531 (0.290 – 0.970)***	0.835 (0.692–1.007)	***0.904 (0.821−0.995)***
Model 2^c^		1	0.727 (0.464–1.137)	0.801 (0.463–1.384)	***0.525 (0.286 – 0.962)***	0.830 (0.685–1.001)	***0.900 (0.816−0.992)***
Stratification by baseline non-communicable diseases status							
Any non-communicable diseases	Number of participants	189	193	113	146	641 (total)	
	Number of cases (%)	27 (14.3)	21 (11.4)	13 (10.6)	10 (6.9)	71 (11.1)	
	Crude	1	0.772 (0.423–1.410)	0.713 (0.346–1.470)	***0.441 (0.206 – 0.944)***	***0.781 (0.621 – 0.984)***	***0.867 (0.770 – 0.977)***
	Model 1^b^	1	0.877 (0.468–1.642)	0.917 (0.418–2.009)	0.626 (0.263–1.486)	0.878 (0.669–1.143)	0.919 (0.797–1.059)
No non-communicable diseases	Number of participants	293	295	157	234	979 (total)	
	Number of cases (%)	26 (8.9)	17 (5.8)	11 (7.0)	10 (4.3)	64 (6.5)	
	Crude	1	0.628 (0.333–1.184)	0.774 (0.372–1.611)	***0.458 (0.216 – 0.971)***	0.799 (0.632–1.010)	0.891 (0.794–1.000)
	Model 1^b^	1	0.597 (0.311–1.147)	0.735 (0.341–1.584)	0.436 (0.187–1.015)	0.784 (0.598–1.019)	0.878 (0.767–1.003)

rJDI: revised Japanese Dietary Index. Odds ratios with statistical significances between the groups are highlighted in bold italics. [a] Odds ratios associated with Q2, Q3, and Q4 were reported in reference to Q1. [b] Model 1 was adjusted for sex, enrollment year, daily caloric intake, body mass index, education level, alcohol use, tobacco use, marriage status, and exercise level [c] Model 2 was adjusted for sex, enrollment year, daily caloric intake, body mass index, education level, alcohol use, tobacco use, marriage status, exercise level, and reported history of any non-communicable diseases (hypertension, hyperlipidemia, coronary artery disease, cerebral vascular accidents, and type II diabetes mellitus). [d] Odds ratio while treating Q1 to Q4 as a continuous trend. [e] Odds ratios per 1-point increment in the rJDI-11 score reported in this column.

As shown in Table 4, we found that higher consumption of fish and shellfish (50.0% vs. 40.7%, p = 0.024), green-yellow vegetables (51.2% vs. 37.8%, p = 0.003), and soybean-derived foods (51.3% vs. 36.3%, p = 0.001) were significantly associated with lower risk of developing geriatric depression. The reduced risk associated with fish and shellfish ceased to be significant after adjusting for Model 1 and Model 2, however the significance remained for green-yellow vegetables and soybean-derived foods.

**Table 4:** Numbers and percentages of participants by geriatric depression status at 70 years of age receiving one point for each dietary item, as well as odds ratio (95% confidence interval) of risk of geriatric depression for each dietary item on the 11-component revised Japanese Diet Index.

		Not depressed (n = 1485)	Depressed (n = 135)				
Dietary Item		n (%)		P value^a^	Crude	Model 1^b^	Model 2^c^
Beneficial	Coffee	915 (61.6)	78 (57.8)				
	Rice	737 (49.6)	77 (57.0)	0.099	1.347 (0.944–1.923)	1.436 (0.960–2.149)	1.469 (0.979–2.202)
	Miso	808 (54.4)	70 (51.9)	0.568	0.902 (0.634–1.284)	0.941 (0.655–1.352)	0.969 (0.674–1.394)
	***Fish and shellfish***	***756 (50.9)***	***55 (40.7)***	***0.024***	***0.663 (0.463 – 0.948)***	0.720 (0.477–1.086)	0.713 (0.473–1.076)
	***Green-yellow vegetables***	***760 (51.2)***	***51 (37.8)***	***0.003***	***0.579 (0.403 – 0.832)***	***0.662 (0.449 – 0.974)***	***0.642 (0.435 – 0.948)***
	Seaweed	754 (50.8)	59 (43.7)	0.116	0.753 (0.528–1.073)	0.890 (0.612–1.294)	0.866 (0.595–1.261)
	Pickles	966 (65.1)	82 (60.7)	0.316	0.831 (0.579–1.193)	0.842 (0.580–1.222)	0.858 (0.590–1.246)
	***Soybean-derived food***	***762 (51.3)***	***49 (36.3)***	***0.001***	***0.541 (0.375 – 0.779)***	***0.605 (0.405 – 0.902)***	***0.590 (0.394 – 0.883)***
	Fruits	752 (50.6)	59 (43.7)	0.123	0.757 (0.531–1.079)	0.932 (0.632–1.374)	0.917 (0.621–1.354)
	Mushrooms	757 (51.0)	58 (43.0)	0.075	0.724 (0.508–1.034)	0.832 (0.573–1.209)	0.803 (0.552–1.168)
Non-beneficial	Meat	735 (49.5)	74 (54.8)	0.237	1.238 (0.869–1.763)	1.066 (0.706–1.608)	1.077 (0.713–1.628)

Dietary items and odd ratios with statistical significances between the groups are highlighted in bold italics. [a] P-values are calculated with Chi-square.

[b] Model 1 was adjusted for sex, enrollment year, daily caloric intake, body mass index, education level, alcohol use, tobacco use, marriage status, and exercise level [c] Model 2 was adjusted for sex, enrollment year, daily caloric intake, body mass index, education level, alcohol use, tobacco use, marriage status, exercise level, and reported history of any non-communicable diseases (hypertension, hyperlipidemia, coronary artery disease, cerebral vascular accidents, and type II diabetes mellitus).

## 4 Discussion

Our study utilized the age-specific prospective cohort NISSIN project to assess the association between adherence to the Japanese diet and the risk of developing geriatric depression after adjusting for the baseline lifestyle and health status. To our best knowledge, this is the first study using a large-scale cohort to identify the association between the adherence to the Japanese diet pattern and the development of geriatric depression among community-dwelling elders.

Overall, we found that the Japanese diet is protective against the development of geriatric depression after adjustment for common confounders. A possible explanation for this is that the Japanese diet pattern is significantly associated with a higher intake of anti-inflammatory nutrients [28]. There is increasing evidence showing that the development of depression is closely associated with the process of neuroinflammation by which pro-inflammatory cytokines may affect the synthesis, release and reuptake of neurotransmitter metabolism related to mood, including dopamine, norepinephrine, and serotonin [29]. In addition, changes in gut microbiota, which is often observed in patients with depression, is also hypothesized to alternate inflammatory status of the microglia which can subsequently cascade from the gut to the central nervous system via the vagus nerve or the immune system [30]. Thus, we propose that the association between higher adherence to a Japanese dietary pattern and reduced risk of geriatric depression as observed in this study might be explained by the modification of pro-inflammatory cytokines by such dietary habits [31].

This hypothesis is further supported by our findings in Table 4 that among all the dietary items analyzed, green-yellow vegetables, soybean-derived products, and possibly fish and shellfish seem to be the major players of this protective effect. Previous studies have shown that antioxidants in vegetables might have beneficial effects against depression [32]. In animal models, antioxidants are found to reverse depressive-like behavior and hippocampal synaptic dysfunction [33]. Thus, the Japanese diet, which is characterized by high vegetable consumption, might prevent and lessen the onset of geriatric depression caused by oxidative stress [34]. Similarly, soybean was found to have antidepressant effects in menopausal women in previous studies and is rich in isoflavonoids, which is known to prevent cognitive decline as well as menopausal vasomotor symptoms [[35], [36], [37], [38]]. Finally, previous studies have argued that the omega-3 PUFA, which is found abundantly in fish, might explain the beneficial effects of fish and shellfish consumption [[39], [40], [41]]. Subsequently, while the role of gut microbiota in the development of depression is still not fully known, we believe it is critical to for future studies to investigate the underlying biophysiological mechanisms.

Yet despite the intriguing nature of the hypothesized diet-microbiome-gut-brain axis, this model remained speculative and other possibilities should be considered. Previous studies have commonly critiqued the influences of residual confounding as well as reversed causality affecting the association of dietary intake and development of depression [42]. Similarly, as shown in Table 2, we noticed that participants with higher adherence to the Japanese diet have higher daily caloric intake (p < 0.0001), possibly due to these participants consuming larger amounts of all the analyzed dietary items. Considering that depression is one of the most common reversible causes of anorexia and weight loss in elderly people, which can be explained by dysregulation of hypothalamic-pituitary-adrenal axis, higher frequency of constipation, and loss of social networks, the dietary intake at baseline which we used to categorize our participant population might as well already be the sequelae of underlying subclinical depression [43]. Furthermore, participants with higher adherence to the Japanese diet were also noted to have significantly higher level of physical activity as shown in Table 1. Effectiveness of exercise in treating and preventing depression was well-established [44,45], and Pearce et al. proposed that exercise might affect the development of depression by multiple pathways including modification of inflammatory response by activation of the endocannabinoid system, improved physical self-perceptions and body image, increased social interactions, and development of coping strategies [46]. As such, while our logistic regression models were adjusted to minimize the confounding effects, the influences of such lifestyle factors might have lingered considering their longitudinal nature and long-lasting effects. Likewise, our logistic regression models with stratifications based on NCDs status at baseline showed prominent modifying effects, as highlighted in Table 3. While the directionality of the association remained the same, the significance of the association grossly diminished after the stratification. This is likely explained by the similarity in pathogenesis between NCDs and depression. HTN was known to be a major risk factor for cerebrovascular pathologies such as white matter lesions, which can potentially lead to late-life depression [47]. DM also shares common biological origins with depression through activation of innate immunity leading to a cytokine-mediated inflammatory response and potential dysregulation of the hypothalamic–pituitary–adrenal axis [48]. Nonetheless, the anti-inflammatory characteristics of the Japanese diet making it protective towards NCDs has already been well established [6,31], as well as the co-occurrence of physical activity and dietary behaviors in psychological distress [49]. Thus given the bi-directional relationship between diet and overall lifestyle as well as physical health, the role of Japanese diet in terms of geriatric depression prevention remained significant from a holistic viewpoint.

Our study, highlighted by being a large-scale age-specific prospective cohort, boasts several strengths. First, by excluding participants with geriatric depression features at baseline and assessing the development of geriatric depression after five years of follow up, our study was able to minimize the effects of pre-existing mood disorders and establish stronger causal relationship than cross-sectional studies. Secondly, the use of scores for the Japanese diet provided more predictive and practical findings, making it easier for individuals to understand and adopt healthier eating habits. While previous cohort studies had assessed the associations between individual nutrients and geriatric depression [50,51], dietary indices and scores of overall diet quality have been found to relate to the risk of disease outcomes more consistently than individual nutrients or foods [52], thus reiterating the importance of our study. Finally, unlike a prior cohort study that similarly focused on the association between dietary scores and depression [13], our study enrolled age-specific participants which suited the purpose of studying geriatric depression, and this allowed us to better focus on a homogenous population with similar physical and psychosocial profiles while minimizing the influence of unexpected confounders.

At the same time, we identified several limitations in our study, the most prominent one being the lack of more comprehensive dietary data mainly due to limitations set by the initial design of the NISSIN project when it launched in 1996. For example, green tea was not included in the questionnaire, possibly because it was such a common drink at the time of assessment [53]. Nonetheless, previous studies had shown that green tea consumption was inversely associated with depressive symptoms [54], and we can reasonably argue that inclusion of this data would have skewed the results toward a stronger association. Similarly, while we were unable to single out beef and pork intake from the overall meat intake, which would also include poultry intake, due to the inherent design of the FFQ. While this evidently would have affected the sensitivity of our findings, populational data has suggested close correlation between the consumption of poultry and other types of meat especially pork [55]. Thus, even without distinguishing the specific type of meat consumption, we believe that the overall meat intake in FFQ should still be rather indicative of beef and pork consumption.

Another major limitation we noticed is that our measurement of geriatric depression is not based on psychiatrist-certified diagnoses. Though a screening tool makes more logistical sense in a large-scale cohort study, this could affect the outcome as patients with NCDs can present with somatic symptoms that mimic depression and subsequently skew the association between diet and depression [13]. While the validity and internal consistency of the Japanese version of GDS-15 have already been established when comparing to the DSM-IV-TR criteria [56], we still took extra precaution in approaching possibly biases, such as stratifying by NCDs status at baseline and considered alternative GDS-15 cutoff scores. Still, incorporating psychiatrist-certified diagnoses will evidently provide more accurate results in follow up studies.

Finally, while our study looked at the association between the Japanese diet and the development of geriatric depression with an overall biophysiological approach, it is worth noting that depression is a dynamic condition that can be heavily influenced by psychosocial factors. Major life-changing events such as the loss of a family member can easily mask the protective effect of a healthy diet or physical well-being and manifest as depressive symptoms. The influence of psychosocial factors on the biophysical pathways of the development of depression remains an important topic that demands further investigation.

## 5 Conclusion

We conducted an age-specific prospective cohort study and analyzed a group of community-dwelling elderly Japanese participants and found that adherence to the Japanese diet appeared to be significantly protective against geriatric depression after adjusting for common confounders. Our study reiterated the importance of adhering to a healthy diet, especially those rich in fish and shellfish, green-yellow vegetables, and soybean-derived products in the elderly population to reduce the risk of developing geriatric depression. From both a public health and individual level, our study provided an evidence-based guidance on dietary recommendations for the elderly population. Adherence to the dietary items highlighted in our study will likely reduce the healthcare burden of geriatric depression within the community, which has the potential to be also applied beyond the Japanese population. We also encourage future studies to consider the possible roles of the diet-microbiome-gut-brain axis in the observed association and unveil the underlying biophysiological mechanism.

## Declaration of competing interest

The authors declare that they have no known competing financial interests or personal relationships that could have appeared to influence the work reported in this paper.
