# Supplementary material for: Adherence to the Japanese Diet is Associated with Lower Risk of Geriatric Depression: A Prospective-Cohort Study Based on the New Integrated Suburban Seniority Investigation (NISSIN) Project
Source: J Nutr Health Aging. 2025 Sep 29;29(11):100689. doi: 10.1016/j.jnha.2025.100689 (PMC12513001; doi:10.1016/j.jnha.2025.100689)
Supplement: Supplementary file 1 [file mmc1.docx]

Supplementary Table 1. Comparison of baseline characteristics between participants without depression at baseline that was exluded from the study versus participants included in the study

| Characteristics | Categories | Included  (n = 1620) | Excluded  (n = 1000) | P value |
| --- | --- | --- | --- | --- |
|  |  | n (%) or Median (1st Quartile - 3rd Quartile) | |  |
| Sex | Female | 793 (49.0) | 497 (49.7) | 0.709^a^ |
| BMI | < 18.5 | 71 (4.4) | 44 (4.4) | 0.757^a^ |
|  | ≥ 18.5 and < 25 | 1198 (74.0) | 727 (72.7) |  |
|  | ≥ 25 | 351 (21.7) | 229 (22.9) |  |
| Exercise Level | Hardly - Never | 610 (37.7) | 398 (39.8) | 0.081^a^ |
|  | Less than once per week | 150 (9.3) | 80 (8.0) |  |
|  | Equal or more than once per week | 860 (53.1) | 519 (51.9) |  |
| Tobacco Use | Never smoker | 890 (54.9) | 559 (55.9) | 0.608^a^ |
|  | ≤ 15 pack-year | 211 (13.0) | 117 (11.7) |  |
|  | > 15 pack-year | 519 (32.0) | 324 (32.4) |  |
| Alcohol Use | Current drinker | 767 (47.4) | 414 (41.4) | 0.291^a^ |
| Education Level | Elementary school or junior high school | 452 (27.9) | 322 (32.2) | 0.064^a^ |
|  | High school or junior college/specialized college | 877 (54.1) | 507 (50.7) |  |
|  | University or higher | 291 (18.0) | 171 (17.1) |  |
| ***Marriage*** | ***Married*** | ***1471 (90.8)*** | ***879 (87.9)*** | ***0.021^a^*** |
| Any Non-Communicable Diseases ^c^ | | 641 (39.6) | 394 (39.4) | 0.932^a^ |
| Daily Sleep Time (hr) | | 7 (6 – 7.5) | 7 (6 - 8) | 0.571^b^ |
| Members Within Same Household | | 1 (1 – 2) | 1 (1 – 2) | 0.638^b^ |
| GDS-15 score | | 3 (1 – 4) | 3 (1 – 4) | 0.385 ^b^ |

GDS-15: Geriatric Depression Scale 15 items

Characteristics with statistical significances between the groups are highlighted in bold italics.
[a] P-values are calculated with Chi-square.
[b] P-values are calculated with Kruskal-Wallis test.
[c] Including any self-reported medical history of hypertension, hyperlipidemia, coronary artery disease, cerebral vascular accident, or type II diabetes mellitus .

Supplementary Table 2. Results of multiple logistic regression models reported in odds ratio (95% confidence interval) while using 6 as cutoff score for depression on GDS-15 at endpoint.

| All Participants | | Q1 | Q2^a^ | Q3^a^ | Q4^a^ | Trend ^d^ | rJDI-11 Score ^e^ |
| --- | --- | --- | --- | --- | --- | --- | --- |
| Number of participants | | 482 | 488 | 270 | 380 | 1620 (total) | |
| Number of cases (%) | | 90 (18.7) | 63 (12.9) | 36 (13.3) | 41 (10.8) | 230 (14.2) | |
| Crude | | 1 | ***0.646 (0.455 – 0.916)*** | 0.670 (0.441 – 1.019) | ***0.527 (0.354 – 0.783)*** | ***0.817 (0.719 – 0.928)*** | ***0.902 (0.845 - 0.962)*** |
| Model 1^b^ | | 1 | ***0.667 (0.465 – 0.955)*** | 0.697 (0.448– 1.085) | ***0.582 (0.370 – 0.918)*** | ***0.843 (0.727 – 0.975)*** | ***0.916 (0.849 - 0.988)*** |
| Model 2^c^ | | 1 | ***0.660 (0.460 – 0.946)*** | 0.682 (0.437 – 1.064) | ***0.575 (0.364 – 0.909)*** | ***0.838 (0.722 – 0.970)*** | ***0.912 (0.845 - 0.984)*** |
| Stratification by baseline non-communicable diseases status | |  |  |  |  |  |  |
| Any non-communicable diseases | Number of participants | 189 | 193 | 113 | 146 | 641 (total) | |
|  | Number of cases (%) | 49 (25.9) | 31 (16.1) | 17 (15.0) | 16 (11.0) | 113 (17.6) | |
|  | Crude | 1 | ***0.547 (0.330 – 0.905)*** | ***0.506 (0.275 – 0.931)*** | ***0.352 (0.191 – 0.649)*** | ***0.710 (0.583 – 0.859)*** | ***0.822 (0.744 – 0.908)*** |
|  | Model 1^b^ | 1 | ***0.586 (0.346 – 0.992)*** | 0.550 (0.284 – 1.064) | ***0.412 (0.203 – 0.836)*** | ***0.746 (0.593 – 0.932)*** | ***0.837 (0.744 – 0.943)*** |
| No non-communicable diseases | Number of participants | 293 | 295 | 157 | 234 | 979 (total) | |
|  | Number of cases | 41 (14.0) | 32 (10.9) | 19 (12.1) | 25 (10.7) | 117 (12.0) | |
|  | Crude | 1 | 0.748 (0.457 – 1.225) | 0.846 (0.473 – 1.514) | 0.735 (0.433 – 1.249) | 0.916 (0.769 – 1.085) | 0.966 (0.886 – 1.055) |
|  | Model 1^b^ | 1 | 0.746 (0.450 - 1.236) | 0.824 (0.448 – 1.515) | 0.721 (0.394 – 1.321) | 0.908 (0.746 – 1.104) | 0.964 (0.871 - 1.066) |

rJDI: revised Japanese Dietary Index.
Odds ratios with statistical significances between the groups are highlighted in bold italics.
[a] Odds ratios associated with Q2, Q3, and Q4 were reported in reference to Q1.
[b] Model 1 was adjusted for sex, enrollment year, daily caloric intake, body mass index, education level, alcohol use, tobacco use, marriage status, and exercise level
[c] Model 2 was adjusted for sex, enrollment year, daily caloric intake, body mass index, education level, alcohol use, tobacco use, marriage status, exercise level, and reported history of any non-communicable diseases (hypertension, hyperlipidemia, coronary artery disease, cerebral vascular accidents, and type II diabetes mellitus).
[d] Odds ratio while treating Q1 to Q4 as a continuous trend.
[e] Odds ratios per 1-point increment in the rJDI-11 score reported in this column
